# Windmill Noise Annoyance, Visual Aesthetics, and Attitudes towards Renewable Energy Sources

**DOI:** 10.3390/ijerph13080746

**Published:** 2016-07-23

**Authors:** Ronny Klæboe, Hanne Beate Sundfør

**Affiliations:** Institute of Transport Economics, Gaustadalléen 21, NO-0349 Oslo, Norway; hbs@toi.no

**Keywords:** wind farms, environmental impacts, noise change

## Abstract

A small focused socio-acoustic after-study of annoyance from a windmill park was undertaken after local health officials demanded a health impact study to look into neighborhood complaints. The windmill park consists of 31 turbines and is located in the South of Norway where it affects 179 dwellings. Simple exposure-effect relationships indicate stronger reactions to windmills and wind turbine noise than shown internationally, with the caveat that the sample size is small (*n* = 90) and responses are colored by the existing local conflict. Pulsating swishing sounds and turbine engine hum are the main causes of noise annoyance. About 60 per cent of those who participated in the survey were of the opinion that windmills degrade the landscape aesthetically, and were far from convinced that land-based windmills are desirable as a renewable energy source (hydropower is an important alternative source of renewables in Norway). Attitudes play an important role in addition to visual aesthetics in determining the acceptance of windmills and the resulting noise annoyance. To compare results from different wind turbine noise studies it seems necessary to assess the impact of important modifying factors.

## 1. Introduction

Globally and in particularly in Northern Europe there is an increasing interest in renewable energy sources, and a number of wind farms have been established. In Norway relatively few wind farms have been established so far, but a number of them are in the planning stage. Wind farm investments can be discounted over a much shorter period, and are thus indirectly subsidized. One of the reasons for the subsidies is industry pressure, as otherwise they will relocate to Sweden where subsidies are in place.

**Figure 1:**
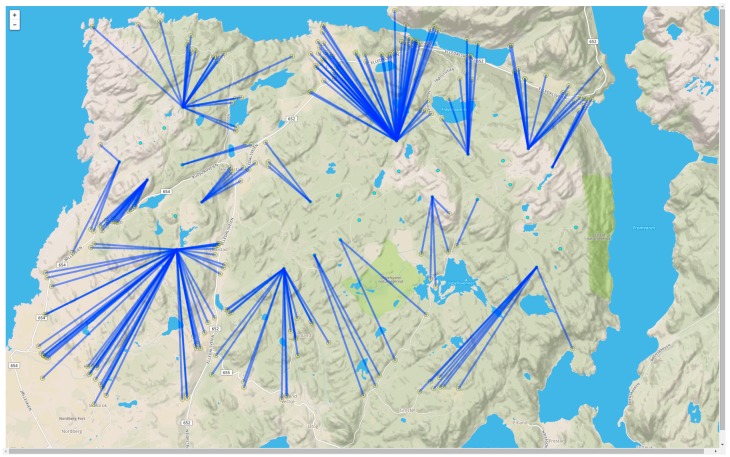
Wind turbines with distance lines to all properties within 2 km of the nearest windmill.

The Lista wind farm was put into operation in 2012. It consists of 31 wind turbines, with 179 properties within a 2 km radius from the closest turbine (Figure 1). Norwegian zoning restrictions are imposed on areas exposed to an evening and night time equivalent wind turbine noise level (L_den_) of 45 dBA and above. The limit for wind turbine noise is thus 10 dBA stricter than the limit for road traffic noise, which is L_den_ 55 dBA. 

The windmills are located behind the shore line on top of a hilly terrain. For some dwellings the windmills are not seen, for others several windmills are in sight and are prominently visible when looking upwards towards the edges of the hill top plateaus.

### International Papers and Results

There are no comprehensive international meta-studies of wind noise similar to those for transportation sources [1,2]. However, there is a paper on the results from a pooled set of Swedish and Dutch studies [3], which can be used to assess whether responses in Norway are stronger or weaker than those found in other European countries. 

Noise sensitivity is an important modifier of noise annoyance and is often described as a personal trait [4,5,6]. Environmental exposures (air pollution, odor, noise, vibrations) are most often little correlated with the respective sensitivities. However, Arezes et al. [7] report higher sensitivity in some more highly exposed areas. On the other hand, Pedersen and Waye [8] report higher sensitivity groups in lower exposed areas.

Land-based wind farms are often placed in sparsely populated rural areas, where background noise levels can be lower and expectations of quietness higher. Rural populations have been reported to be more annoyed than urban populations. However, it is not clear whether this is due to the lower background levels [9]. Whereas the sound energy from the wind turbines can be modest as compared with the noise levels urban populations regularly are exposed to, the changes to the soundscape relative to the before situation can be significant. Wind farms can be regarded as an undesirable industrialization of agricultural, forestry and natural landscapes. People prefer continuity [10] and resist abrupt changes to their living environment. Kageyama et al. [11] find no impact of wind turbine noise on health, but report that visual annoyance and noise sensitivity are associated with poor health. They interpret the impact of these moderators as indicative of persons that are sensitive to changes in their homeostasis. Pedersen and Waye [8] discusses coping activities. Given the small sample size, lack of before data and the limited purpose of the study, we have not analyzed the potential impact of such factors.

It is well known that the visual landscape can modify noise annoyance, and a pleasing visual environment can be utilized to reduce noise annoyance [12,13,14,15]. For wind farms, the visual aspects are known to increase noise annoyance. We distinguish between direct visual effects, the visibility and visual intrusion of having the windmills in view from the respondent dwelling, and windmills as negative elements of the visual landscape. Rotating windmill blades cause visual disturbances by intercepting sun light, thereby causing moving shadows, and light flashes from reflecting sun light [16]. A combination of acoustic and visual exposure leads to a more negative assessment of the windmills [8,17]. At northern latitudes, with the sun low on the horizon, shadow casts can be more prominent. In addition to the visibility of the windmills, attitudes towards windmills can affect the annoyance responses [7,9]. 

What residents expect of the landscape can also be important [18,19], as well as how authorities manage the introduction of new sound sources [20,21]. There are a number of studies showing that noise annoyance is higher when new noise sources are introduced, or when there is a sharp increase in noise exposure [22]. The most prominent health effect associated with wind turbine noise is the generated noise annoyance reactions [23,24,25,26]. 

Two thirds of the energy in Norway comes from renewable sources—mainly hydropower. This is four times higher than e.g., Germany and ten times higher than in The Netherlands. To meet increasing demands for energy, many Norwegians would prefer new or expanded hydropower installations. Many countries have less desirable alternatives (solar power, coal, gas and/or nuclear powered installations) due to land use, intermittency, local air pollution, global emissions and security. 

## 2. Method

After the establishment of the wind farm in 2012, the residents complained about the noise from the wind turbines, and how persistent and bothersome the noise turned out to be. A socio-acoustic study with deadline the end of 2015 was required by the local health authority to look into possible causes for local noise complaints.

It is not an uncommon scenario that authorities fail to respond until new projects are met with more resistance than expected up front. Involving researchers late in the process and after the infrastructure is already in place is far from the ideal situation with respect to research design. The current study is thus an after study, undertaken at a single site. The lack of before study data limits the analyses and type of conclusions that can currently be drawn from the results. Pooling the survey data from the Lista study with before-after results from additional study areas could make it possible to extract common and site specific factors. However, there are as yet no plans for multi-site before-after studies.

The study is a socio-acoustic study where responses to questionnaire items are analyzed relative to exposure indicators in the form of the calculated outdoor noise level at each respondent dwelling, and the linear distance to the closest wind turbine. The study was undertaken in September/October 2015. 

### 2.1. Questionnaire

The questionnaire was introduced as a study of the living environment. Given the local conflict and community awareness of a pending survey, the introduction fails to mask the “real” purpose of the survey. 

However, framing the survey as a general survey of living quality avoids some of the focusing effects of a narrow wind turbine noise approach, and imposes a more general frame of reference. The survey was voluntary, with a response rate of 38 per cent which was as expected for this type of survey. 

The questionnaire contains sections on the living environment, health, annoyances, non-acoustic modifiers such as visual aesthetics, attitudes, and noise sensitivity. The noise annoyance questions build upon the work by [27,28], but has separate questions for the outdoor and indoor situations. The questions used were:

“When thinking about the last 12 months, how annoyed are you by noise from windmills when you are inside your own dwelling? (five-point scale: Extremely annoyed, Very annoyed, Moderately annoyed, Slightly annoyed, Not annoyed, Not relevant)”

“When thinking about the last 12 months, how annoyed are you by noise from windmills when you are right outside of your own dwelling? (five-point scale: Extremely annoyed, Very annoyed, Moderately annoyed, Slightly annoyed, Not annoyed, Not relevant)”

The response category “*Not relevant*” was merged with ”*Not annoyed*” after ensuring that the respondent did not live outside the designated study area, and was included in the sampling frame. 

#### Sensitivity to Vibration and Noise

It is known from noise research that noise sensitivity is a personal trait that is important for evaluation of noise annoyance [29]. The perceived noise level must be doubled before a non-sensitive person reacts as strongly as a person who is highly noise sensitive. From environmental studies, we also know that people who say they are noise sensitive also react more strongly to air pollution [30,31]. The sensitivity to environmental exposure has both common and exposure specific components. Within stress and cognitive activation theory (CATS), sensitivity, and sensitization (including cross-sensitization) to chemicals play a major role [32,33,34,35,36]. 

Several authors [37,38] advocate the use of multi-item noise sensitivity scales. However, the number of questions need to be kept at a minimum to reduce response time. We find that questionnaires requiring more than 15–20 min, result in higher rates of attrition. We consequently employ a single noise question on the degree of noise sensitivity which in our experience works well. The response categories are “very”, “partly”, “not” and “don’t know”. The latter category was merged with “not” sensitive. With respect to the health effects one line of thought is that noise exposure causes stress-reactions that over time have adverse health effects. However other models are possible [4,39,40]. 

### 2.2. Noise Calculations

The noise calculations were undertaken using CADNA [41]. In separate project the calculated values were controlled with a measurement regime [42]. Since the noise measurements, resulted in estimates that were not significantly different from the calculated values (+/−1 dBA), we use the calculated values. 

In Norwegian regulations daytime is counted as 06:00 a.m. to 18:00 p.m., the evening period from 18:00 p.m.–22:00 p.m. and night time 22:00 p.m. to 06:00 a.m. The evening and night weighting adds 6.4 dB. A wind turbine noise source emitting L_Aeq,1h_ = 38.6 each hour of the diurnal cycle, will thus satisfy the 45 dBA limit.

The study population was defined as all residents 18 years or older living closer than 2 km from the nearest wind turbine. The population was extracted by a three stage process. All 179 properties within 2 km were extracted from the national property register. All 240 identified owners of these properties were thereafter obtained from the municipality ownership records. Since the population was small, every member of the household had the opportunity to answer. Only a few respondents made use of this opportunity. 

### 2.3. Analyses

#### 2.3.1. Descriptive Analyses

For simple tables and bar-charts as a function of noise exposure, we analyze responses in each of four noise exposure intervals (<36.9 dBA, 37–39.9 dBA, 40–42.9 dBA, 43+ dBA) containing about the same number of people.

Simple stacked bar charts are used to illustrate the proportion of respondents that hold different levels of belief in different statements concerning windmills and renewable energy sources. Stacked bar charts for each of the noise exposure intervals are used for noise sensitivity.

Attrition is especially problematic when the attitudes of respondents differ from non-respondents. Noise calculations for all potential respondents were available, and it was thus possible to calculate the attrition rate for each of the noise exposure intervals. This makes it possible to see whether only the groups most affected respond, or whether the response rate is uniform—not dependent on the level of wind turbine noise the respondent is exposed to.

#### 2.3.2. Exposure–Effect Modelling

Ordinal logit models [43,44,45] were used to estimate exposure–effect relationships between noise exposure (L_den_) and annoyance responses. They belong to the same class of models as grouped regression models [2]. SPSS version 22 (IBM Corp., Armonk, NY, USA) [46] was used for the estimation of all models.

To control for noise sensitivity, the indicator was added as an ordinal modifying variable to models for annoyance when outside and inside dwelling as a function of noise exposure. Since there were more noise sensitive persons in the second highest noise exposure interval, we estimated exposure–effect relationships given a more homogenous spread of noise sensitivity. We also show the difference between exposure–effect relationships with and without correction for noise sensitivity. Klæboe et al. [47] give a short but detailed description on how to adjust for sensitivity, and how to calculate confidence bands. See also a web-based tool for calculating exposure-effect relationships from statistical output [48].

To assess the potential impact of visual aesthetics, we run models where we control for people’s perception of windmills as an aesthetic problem. Since the number of respondents are limited, the models are kept simple. 

Without a pre-study it is not possible to determine accurately whether attitudes towards renewable energy sources cause higher or lower levels of annoyance or vice versa (reverse causality). We estimate a model where attitudes towards renewables is introduced as a modifying factor—bearing part of the responsibility for the annoyance reactions. This would not be correct if the causality is reverse. 

However, if it is the respondents’ personal experience with windmills and associated noise problems, that turn them against renewable energy sources, we would expect that those most affected by wind turbine noise were most critical of renewables. To check whether this is the case, we compare the attitudes to renewables in each of the wind turbine noise exposure intervals. Stacked bar charts for each of the noise exposure intervals are used for attitudes towards renewables.

Only 39% of the sample use their dwelling the whole year. About 60% list that the properties are only used in part of the year. Since residents might have higher expectations with regards to the sound environment when taking a vacation or using the dwelling for recreational purposes, we also estimate an ordinal logit model controlling statistically for type of dwelling.

### 2.4. Assessment of Limit Values

Different noise sources, generate different levels of annoyance. Norwegian land use regulations impose zoning restrictions around air ports, shooting ranges, windmills, and other point sources and along road and rail infrastructure. To establish yellow and red noise zones that give equal protection, an adjustment factor is added to the noise level so that zones along roads and railway lines, air ports, industry sites or windmills provide the same protection. Using data from the Norwegian façade insulation studies [49,50] it is shown that 21% of the population is extremely + very annoyed by road traffic noise at the yellow zone limit of 55 dBA. New infrastructure leading to outdoor noise levels exceeding the limit need to ensure that affected dwellings have access to a quiet side, or sheltered areas.

### 2.5. Comparisons against International Results

**Figure 2:**
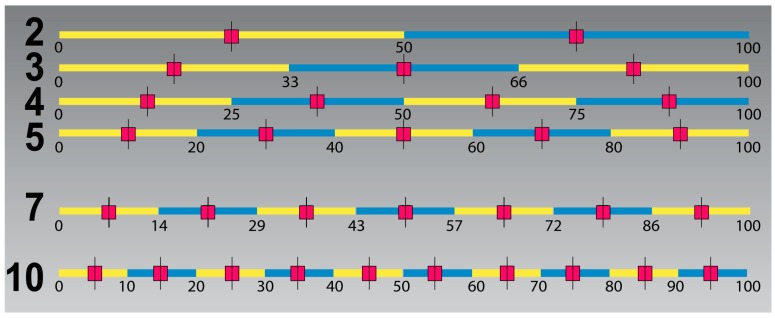
How noise annoyance scales with a different number of scale points partition a continuous noise annoyance indicator going from 0 to 100 given that the scale partitions are equal in size and cover the whole range of responses.

When comparing results from international studies of noise reactions, one of the problems has been the use of scales with different numbers of scale points. Given that each scale divides a continuous noise annoyance indicator from 0 to 100 where 0 indicates no annoyance at all, and 100 indicates totally annoyed [51], the dividing points and mean scores within each interval can be depicted, see Figure 2.

We are comparing against results by Janssen [3] who for compatibility reasons, report their results according to a seven-point scale where the proportion of highly annoyed are more than 72% annoyed, %HA_72_. We both use and reports results according the international 5-point scale, where the proportion of respondents who chose the upper two categories is %HA_60_. From Figure 2 it can be seen that the dividing point lies in the middle between 50% and 72% continuous annoyance on a scale from 0 to 100. See also [51].

We thus expect our exposure–effect relationship for %HA_60_ to lie midway between the curves for %A_50_ and %HA_72_ and we can thus interpolate between the curves. We use this result as an aid when comparing our results against those from Janssen et al. [3].

## 3. Results

### 3.1. Fewer Respondents at Lowest Noise Exposure Interval

**Figure 3:**
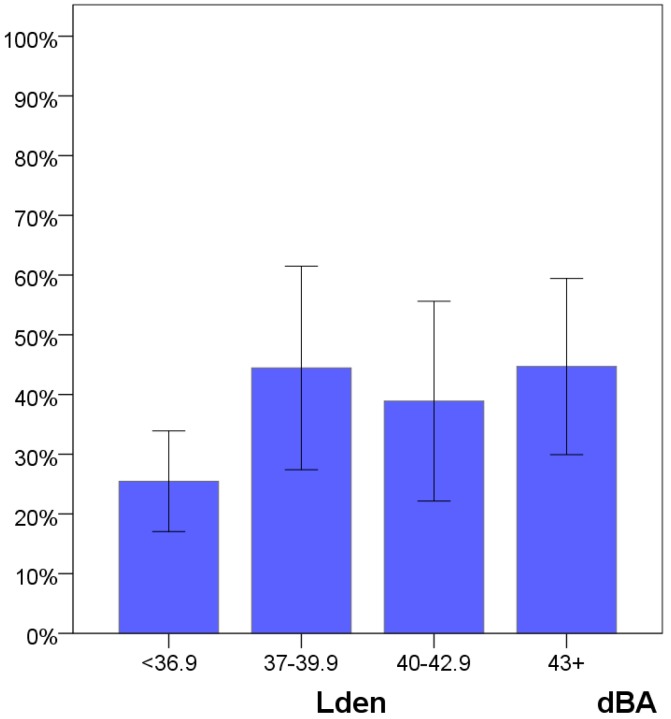
Response rate (blue) as a function of noise exposure (four intervals). Lista wind farm 2015. (Error bars denote 95% confidence intervals).

The response rate was 38% which was as expected when using this type of survey instrument. We have charted non-response as a function of noise exposure. Figure 3 reveals that we have received a lower proportion of responses when noise levels are low. This will affect results where we do not control for noise exposure, resulting in somewhat higher annoyance rates. For exposure–effect relationships where we control for noise exposure level this is a lesser problem. 

If the reduced response rate for the lowest noise interval signifies that people who are less affected by environmental exposures refrain from answering, control for noise exposure solves only part of the problem and we will register higher levels of annoyance than for the population as a whole. There are no obvious signs of such an effect in that response rate in the two most noise exposed groups are no higher than in the second lowest group. 

### 3.2. Attitudes

**Figure 4:**
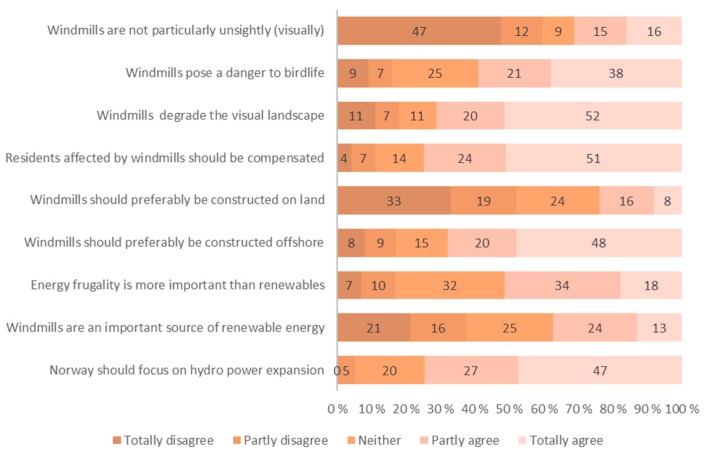
Respondents who agree or disagree to a set of prepared opinion statements. General context. *n* = 90.

**Figure 5:**
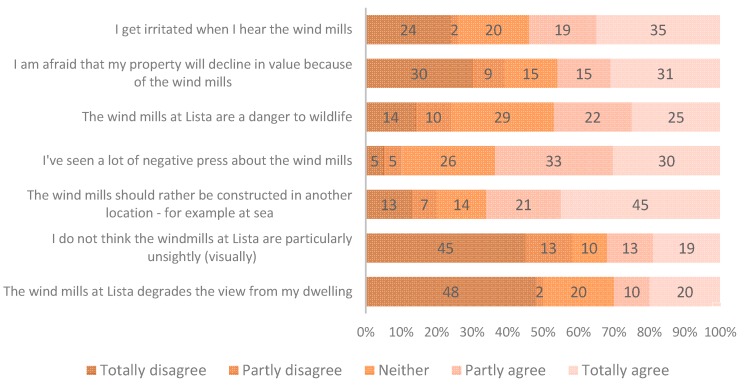
Respondents who agree or disagree to a set of prepared opinion statements. Local (Lista) context. *n* = 90.

A number of statements with respect to public acceptance and resistance to wind farms were used to elicit people’s attitudes towards the windmills and to renewable energy sources, see Figure 4 and Figure 5. Likert scales, where the respondents are asked how much they agree or disagree with the statements, were used.

About 70% agree that windmills degrade the visual landscape. The majority of the respondents (74%) agree that Norway should focus on hydro power expansion. About 60% of the respondents disagree with the statement that the windmills are not particularly unsightly—and are thus dissatisfied with the visual aesthetics, and the contrast between the windmills and the landscape.

### 3.3. Noise Sensitivity

**Figure 6:**
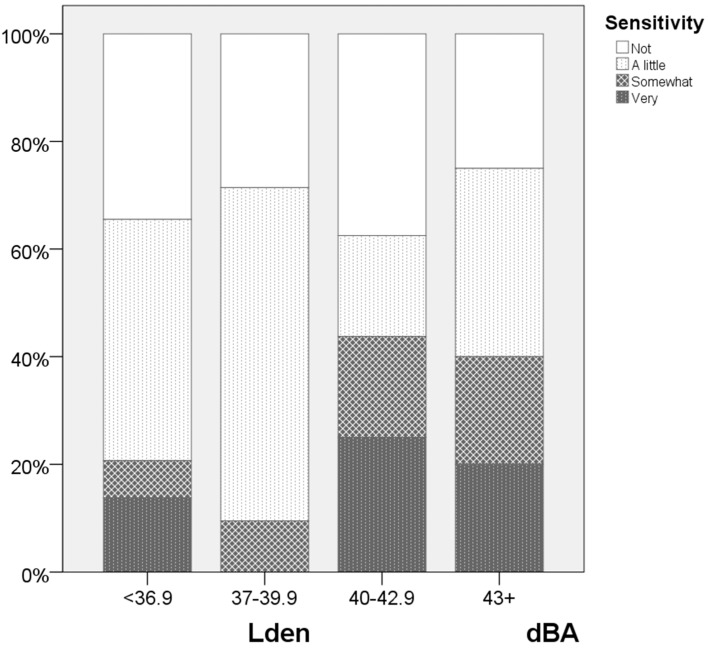
Noise sensitivity by noise exposure. Stacked bar chart as a function of noise exposure. Four exposure intervals.

Given the low number of respondents, it is not to be expected that we have the same proportion of noise sensitive in each of the exposure intervals. Results show a higher proportion of noise sensitive respondents especially in the next highest exposure interval (40–42.9 dBA), see Figure 6. The skewed distribution will affect the exposure-effect relationships and elevate the level of annoyance as a function of exposure. We consequently control for noise sensitivity in the analyses, see however the discussion section.

### 3.4. Visual Aesthetics

**Figure 7:**
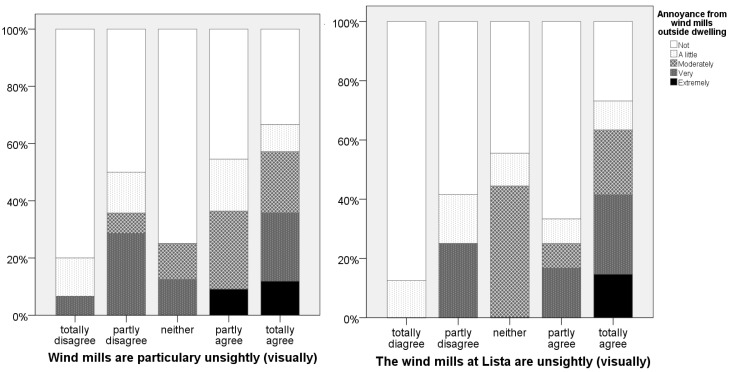
Level of agreement with the statement that windmills are a visual blight in general (**left panel**) and at Lista in particular (**right panel**). *n* = 90.

Many respondents, report that the windmills in general and Lista in particular, are a visual blight and detrimental to the aesthetic landscape, see Figure 7. We consequently control for aesthetic considerations in addition to whether the wind mills are visible or not when establishing exposure–effect relationships.

### 3.5. Exposure-Effect Relationships

#### Simple Model for Wind Turbine Noise Annoyance by L_den_

**Figure 8:**
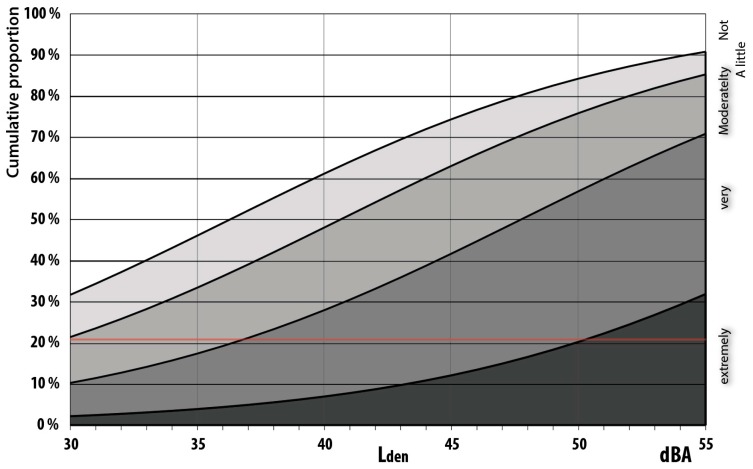
Exposure–effect relationships for annoyance right outside the dwelling from wind turbine noise as a function of evening and night weighted equivalent noise levels (Lden). *n* = 87.

Simple exposure–effect relationship summarizes wind turbine noise annoyance as a function of noise exposure without control for noise sensitivity, attitudes towards visual aesthetics and renewables, see Figure 8 and Appendix Table A1.

About 21 per cent of the population are extremely and very annoyed right outside their apartment by road traffic noise at 55 dBA. From Figure 8 it can be seen that this level of annoyance right outside the dwelling is reached at a wind turbine noise level of L_den_ 37 dBA, numerically 18 dBA lower than for road traffic and 8 dBA lower than the current zone limit (45 dBA). However, we need to adjust for the skewed distribution of noise sensitive respondents, see Figure 6.

### 3.6. Adjusting Relationships for Noise Sensitivity

**Figure 9:**
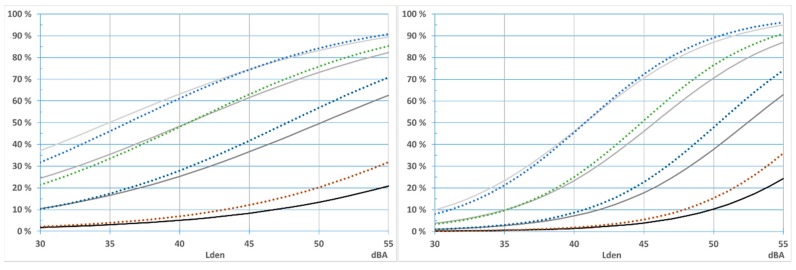
Exposure–effect relationships for annoyance right outside dwelling (**left panel**), and when indoors (**right panel**). Curves before (dotted lines) and after correction for noise sensitivity. *n* = 87.

We usually treat noise sensitivity as an individual trait that is not correlated with noise exposure. We consequently control statistically for noise sensitivity, see Appendix Table A2 (annoyance outdoors) and Table A3 (annoyance indoors). This separates the effect of wind turbine noise from that of noise sensitivity and shows how the exposure–effect relationships would look like with the same proportion of noise sensitive respondents for each exposure interval, see Figure 9. 

When controlling for noise sensitivity the line of highly annoyed crosses the 21% line slightly to the right of the uncontrolled line, at about 38 dBA, and the malus is consequently estimated to be 17 dBA instead of 18 dBA, see Figure 9 left panel. 

### 3.7. Comparisons of Limit Values

We have chosen to compare the exposure–effect relationships from the Lista study with the combined results from several Swedish and Dutch studies [3]. Since the Norwegian results are derived from an annoyance scale with five categories (ISO TS1999) with a 60% cut-off whereas the Swedish/Dutch study reports results converted to a scale with seven categories with a 72% cut-off, we need to make these comparable, see Figure 2.

**Figure 10:**
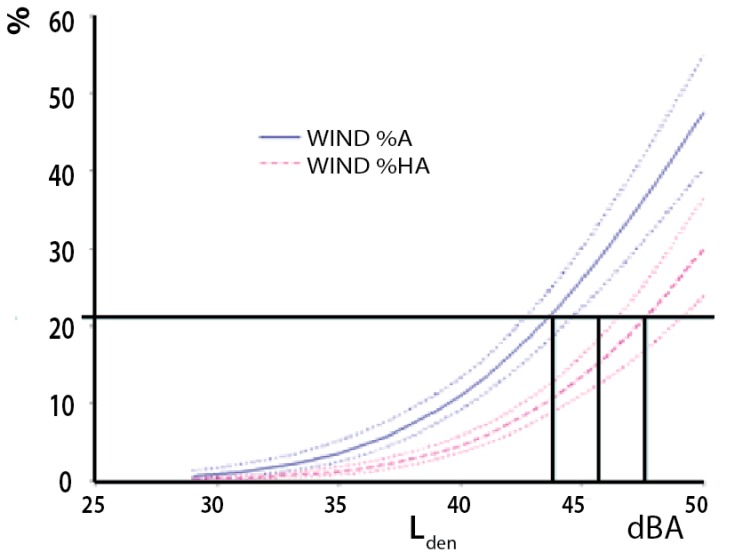
Exposure–effect relationships for wind turbine noise annoyance when outside the dwelling Janssen et al. [3]. (21%-line and drop-lines added by us). This figure is reproduced with the permission of Acoustical Society of America.

In Norway about 21% are extremely and very annoyed (%HA_60_) when road traffic is 55 dBA (start of yellow zone). The curves for %HA_60_ for the Swedish and Dutch relationships are located about midway between the curves for %A_50_ and %HA_72_ and crosses the 21% line at about 46 dBA, see Figure 10.

This indicates that 21% highly annoyed when outside (%HA_60_) is reached at 46 dBA, and that the combined Dutch and Swedish studies result in an estimated malus relative to road traffic noise of about 9 dBA. These results are in accordance with 10 dBA malus according to current Norwegian noise regulations. As previously shown community reactions in Lista are significantly stronger than that with a malus of 17–18 dBA, see Figure 9 left panel.

### 3.8. Aesthetics as a Modifying Factor

We have estimated a model where we simply control whether one or more windmills are visible or not, see Appendix Table A4. The visibility of the windmills is associated with being able to identify the noise source, visual disturbances from moving shadows and light reflections from the rotating rotor blades, and aesthetic aspects. Having no windmills in view is associated with a significant and substantial reduction in annoyance. Given the hilly terrain it is possible to be affected by noise from close-by visually shielded wind turbines. 

**Figure 11:**
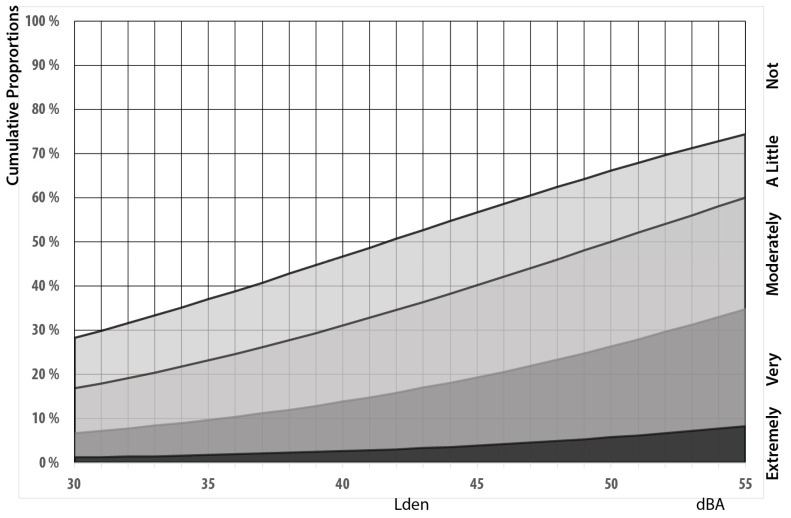
Exposure–effect relationships for people who are annoyed at outside dwelling as a function of L_den_. Curves for respondents who see one windmill, but don’t regard windmills as degrading the visual landscape. *n* = 87.

Respondents who report that they consider windmills a visual blight (in general or at Lista in particular) are significantly more annoyed. The effect is substantial and in size equivalent to 11 dBA, see Appendix Table A5. More than half of the respondents are of the opinion that windmills are detrimental to the visual landscape. Of those who are amenable to the visual aesthetics, the exposure-effect relationships are similar to those we found in Janssen et al. [3], with the curve for highly annoyed crossing the 21% line at 46 dBA resulting in an estimated malus of 9 dBA, see Figure 11.

### 3.9. Attitudes towards Renewable Energy Sources

**Figure 12:**
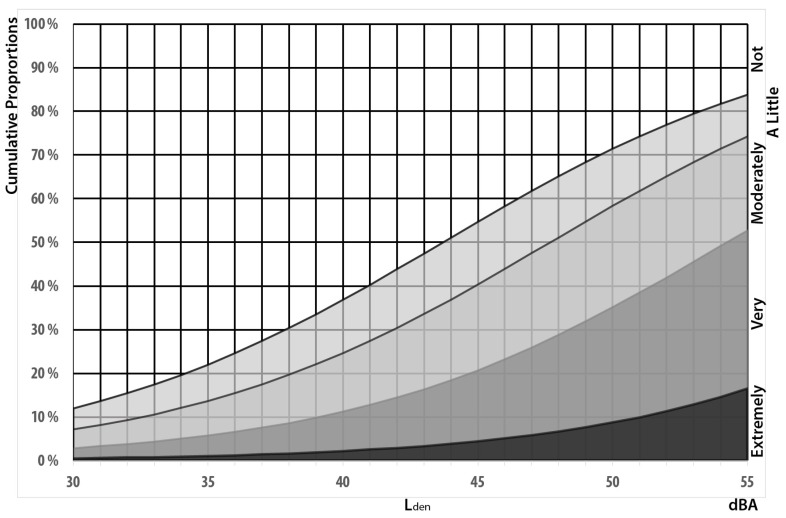
Exposure–effect relationships for annoyance from wind turbine noise outside dwellings for residents who are indifferent or do not agree with the statement that Norway should rather prioritize hydropower (*n* = 87).

A model controlling for whether people agreed totally or in part that Norway should prioritize hydropower versus those who were ambivalent or disagreed indicate that respondents’ annoyance reactions are strongly colored by their attitudes towards renewables—about 10 dBA, see Figure 12 and Appendix Table A6. 

There is only a correlation of about 0.40 between the degree of agreement whether Norway should prioritize hydropower, and whether windmills destroy the landscape. Noise sensitivity was neither correlated with attitudes towards hydropower nor responses to whether the windmills were visually “degrading” the landscape. Exposure–effect relationships for people who disagree that Norway should develop hydropower instead See Figure 7, indicates that a limit value of about 45 dBA, a malus of 10 dBA vs road traffic, for this current minority provides the same level of protection as the limits for road traffic offer the population as a whole. 

### 3.10. Exposur-Effect Relationships Distance

The Norwegian terrain is hilly and there can be substantial vertical distance between the windmills and the dwellings. Sound propagation may thus differ from situations in Denmark or the Netherlands where sound propagates more horizontally. 

**Figure 13:**
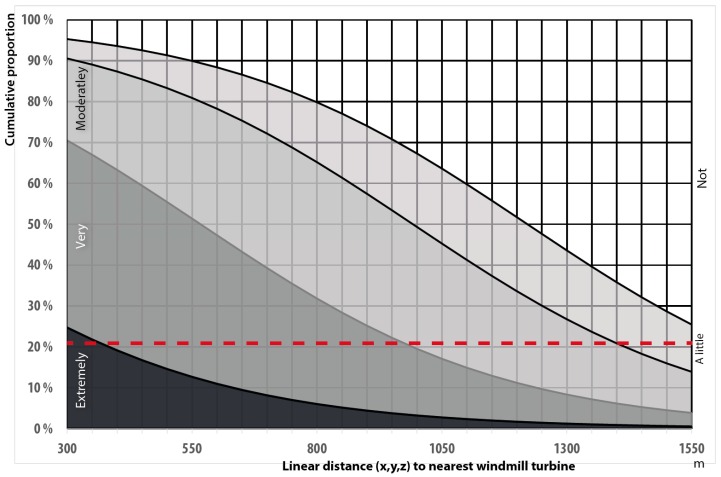
Exposure–effect relationships for annoyance right outside the dwelling from wind turbine noise as a function of linear distance. *n* = 87.

From the exposure–effect relationships as a function of distance, see Figure 13, we have similar results as for exposure–effect relationships based on L_den_, namely that the residents react stronger than expected. According to the estimated results, the windmills need to be located as far as 1 km away from the dwellings before the annoyance levels become acceptable.

### 3.11. Influence of Monetary Benefits

Three quarters of the respondents were in favor of monetary compensation for wind turbine noise. However, we found no significant impact of economic compensation on their degree of annoyance. Part of the explanations could be that not all land owners receiving compensation were the actual users of the property. 

## 4. Discussion

When dealing with annoyance reactions to transportation noise, the acoustic environment is usually adverse. Attitudes and modifying factors compete against a strong negative laden perceptual experience and active disruptions of ongoing activities. When dealing with annoyance from windmills the situation is usually quite different, and the exposure levels are substantially lower. It is not surprising that this allows the role of attitudes and effect modifiers to play a greater role. 

One question is whether these attitudes are stable or transitionary. A significant proportion of the local population are currently in opposition to reigning policies facilitating investments in wind farm. Those in favor of windmills could speculate that one of the reasons is that wind farm are a recent development, and will be more accepted as time goes on. However, the opposite is also possible. Opponents could argue that the energy-mix in Norway is different, the love of recreational areas, and un-touched nature areas higher, and that one can expect a steady opposition to wind farms. 

Strong national and international interests argue that building wind farm is absolutely necessary to achieve renewable targets and degradation of the local environment a necessary evil. Local interests argue that the establishment of wind farm destroys the local environment and that wind farm should be located off-shore or, for Norway in particular, replaced by hydropower. These are questions that are difficult to address scientifically, and are subject to political debate and political differences. 

Since the energy mix, topology, attitudes and land use is different in different countries, it is doubtful whether meaningful comparisons of people’s reactions can be made from acoustic factors alone. In the ordinal logit model, it is the logarithm of the odds of being highly annoyed that is a function of noise exposure and modifying factors. In practice this means that the values on the modifying factors serve as multipliers and that the same noise exposure level will result in different levels of annoyance depending on site location, and the associated modifying factor values. This makes it difficult to compare limit values for wind turbine noise on the basis on acoustics and measurement standards alone. Since attitudes towards renewables are subject to change, net displacement factors [26] are unlikely to be stable. Standard questionnaire items assessing the impact of non-acoustic factors may be required to separate the impact of different factors, and to compare results from different studies.

### Reverse Causality

**Figure 14:**
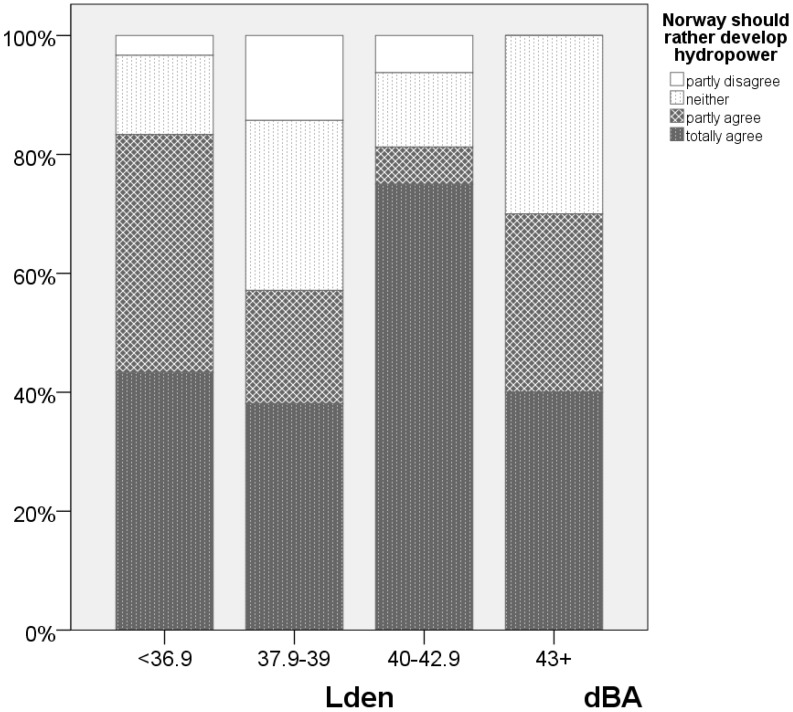
Level of agreement with the statement that Norway should rather develop hydropower. Respondents exposed to different levels of wind turbine noise (four exposure intervals). *n* = 87.

Since the study is a simple after-study it is not possible to determine directly whether people’s annoyances are influenced by their attitudes towards windmills, or vice versa, whether they have changed their attitudes towards windmills as a renewable energy sources as a consequence of their own experiences with wind turbine noise. However, a simple stacked bar diagram showing the residents’ attitudes towards windmills as a renewable energy source indicates that there is no clear correlation between these attitudes and wind turbine noise exposure, See Figure 14. In the reverse causality situation, we would expect the attitudes to show a clear influence of noise exposure. The results support a straight forward link between attitudes towards renewables as a modifying factor with respect to windmill noise annoyance.

## 5. Conclusions

Noise from will mills is considered 17–18 dBA worse than road traffic noise–if we take the results at face value and disregard the large impact on annoyance from non-acoustic factors. This is within the range of 11–26 reported by Michaud et al. [26].

However, our results clearly show that noise annoyance depends strongly on separate non-acoustic factors. Visual and aesthetic factors play a large role together with attitudes towards wind farms as a renewable source of energy. Given the limited number of respondents, it is not possible to separate these factors, but the moderate correlations indicate that the opposition to the wind farm is not monolithic but the result of multiple individually contributing factors.

## Appendix. Parameter Estimates

**Table A1a:** Estimated parameters of a simple exposure–effect relationship between noise exposure (Lden) and wind turbine noise annoyance without control for sensitivity, visual aesthetics or attitudes. *n* = 87.

		Estimate	Std. Error	Wald	df	Sig.
Threshold	(awmno = 1)	4.345	1.380	9.917	1	0.002
	(awmno = 2)	4.856	1.398	12.070	1	0.001
	(awmno = 3)	5.700	1.427	15.962	1	0.000
	(awmno = 4)	7.331	1.494	24.088	1	0.000
Location	Lden	0.118	0.035	11.090	1	0.001

**Table A1b:** Estimated covariance matrix.

		Threshold				Location
		(awmno = 1)	(awmno = 2)	(awmno = 3)	(awmno = 4)	Lden
Threshold	(awmno = 1)	1.904	1.917	1.940	1.968	0.048
	(awmno = 2)	1.917	1.954	1.974	2.000	0.049
	(awmno = 3)	1.940	1.974	2.036	2.056	0.050
	(awmno = 4)	1.968	2.000	2.056	2.231	0.051
Location	Lden	0.048	0.049	0.050	0.051	0.001
Link function: Logit.						

**Table A2a:** Estimated parameters of the relationship between noise exposure (L_den_) and wind turbine noise annoyance outside dwelling, with control for noise sensitivity. *n* = 86.

		Estimate	Std. Error	Wald	df	Sig.
Threshold	(awmno = 1)	7.269	2.290	10.075	1	0.002
	(awmno = 2)	7.880	2.314	11.598	1	0.001
	(awmno = 3)	8.934	2.357	14.363	1	0.000
	(awmno = 4)	10.821	2.442	19.629	1	0.000
Location	Lden	0.215	0.057	14.504	1	0.000
	(sensitive = 1)	−1.398	0.656	4.541	1	0.033
	(sensitive = 2)	−1.449	0.629	5.307	1	0.021
	(sensitive = 3)	0.384	0.764	0.254	1	0.615
	(sensitive = 4)	0 ^a^			0	

^a^ This parameter is set to zero because it is redundant.

**Table A2b:** Case processing summary.

		*N*	Marginal Percentage
Annoyance outside apartment	Not	40	46.5%
	A little	10	11.6%
	Moderately	14	16.3%
	Very	16	18.6%
	Extremely	6	7.0%
How sensitive	Not	27	31.4%
	A little	36	41.9%
	Somewhat	11	12.8%
	Very	12	14.0%
Valid		86	100.0%
Missing		4	
Total		90	

**Table A3a:** Estimated parameters of the relationship between noise exposure (L_den_) and indoor wind turbine noise annoyance with control for noise sensitivity. *N* = 87.

		Estimate	Std. Error	Wald	df	Sig.
Threshold	(awmni = 1)	7.520	2.297	10.720	1	0.001
	(awmni = 2)	8.550	2.339	13.358	1	0.000
	(awmni = 3)	9.923	2.392	17.215	1	0.000
	(awmni = 4)	11.587	2.495	21.575	1	0.000
Location	lden	0.206	0.056	13.697	1	0.000
	(sensitive = 1)	−0.873	0.698	1.568	1	0.211
	(sensitive = 2)	−1.473	0.691	4.550	1	0.033
	(sensitive = 3)	0.116	0.796	0.021	1	0.885
	(sensitive = 4)	0 ^a^			0	

^a^ This parameter is set to zero because it is redundant.

**Table A3b:** Case Processing Summary.

		*N*	Marginal Percentage
Annoyance inside apartment	Not	51	59.3%
	A little	13	15.1%
	Moderately	13	15.1%
	Very	7	8.1%
	Extremely	2	2.3%
How sensitive	Not	27	31.4%
	A little	36	41.9%
	Somewhat	11	12.8%
	Very	12	14.0%
Valid		86	100.0%
Missing		4	
Total		90	

**Table A4:** Estimated parameters of the relationship between noise exposure (L_den_) and wind turbine noise annoyance with control for sensitivity, and number of windmill turbines in view. *N* = 86.

		Estimate	Std. Error	Wald	df	Sig.
Threshold	(wmno = 1)	0.564	1.750	0.104	1	0.747
	(wmno = 2)	1.238	1.755	0.498	1	0.481
	(wmno = 3)	2.314	1.767	1.714	1	0.190
	(wmno = 4)	4.187	1.818	5.305	1	0.021
Location	lden	0.067	0.041	2.696	1	0.101
	(wmt_inview = 0)	−2.107	0.780	7.299	1	0.007
	(wmt_inview = 1)	0.208	0.711	0.086	1	0.770
	(wmt_inview = 2)	0 ^a^			0	
	(sensitive = 1)	−1.488	0.690	4.645	1	0.031
	(sensitive = 2)	−1.863	0.685	7.401	1	0.007
	(sensitive = 3)	0.046	0.786	0.003	1	0.953
	(sensitive = 4)	0 ^a^			0	

^a^ This parameter is set to zero because it is redundant.

**Table A5:** Estimated parameters of the relationship between noise exposure (L_den_) and wind turbine noise annoyance with control for whether windmills are perceived as visually degrading (in general, or at site location). *N* = 86.

Parameter Estimates						
		**Estimate**	**Std. Error**	**Wald**	**df**	**Sig.**
Threshold	(wmno = 1)	4.457	1.382	10.398	1	0.001
	(wmno = 2)	5.049	1.405	12.914	1	0.000
	(wmno = 3)	6.020	1.444	17.380	1	0.000
	(wmno = 4)	7.759	1.523	25.943	1	0.000
Location	lden	0.136	0.036	14.245	1	0.000
	(vis_degrading = 0)	−1.548	0.494	9.820	1	0.002
	(vis_degrading = 1)	0 ^a^			0	

^a^ This parameter is set to zero because it is redundant. Link function: Logit.

**Table A6:** Estimated parameters of the relationship between noise exposure (L_den_) and wind turbine noise annoyance with control for whether people agree or are ambivalent/disagree whether Norway should prioritize hydro power. *N* = 87.

		Estimate	Std. Error	Wald	df	Sig.
Threshold	(wmno = 1)	4.9073657	1.419	11.961	1	0.001
	(wmno = 2)	5.4869193	1.443	14.462	1	0.000
	(wmno = 3)	6.4365440	1.483	18.842	1	0.000
	(wmno = 4)	8.1681559	1.563	27.321	1	0.000
Location	lden	0.1453391	0.037	15.264	1	0.000
	(ProH = 0)	−1.4454090	0.512	7.975	1	0.005
	(ProH = 1)	0 ^a^			0	
Link function: Logit.						

^a^ This parameter is set to zero because it is redundant.
